# Adequate Iodine Intake among Young Adults in Jiangsu Province, China Despite a Medium Iodine Knowledge Score

**DOI:** 10.3390/ejihpe10010040

**Published:** 2020-03-09

**Authors:** Yifan Jin, Xiaoqin Luo, Zheng Feei Ma, Zihan Dong, Richard Carciofo, Xinli Li, Sheila Skeaff

**Affiliations:** 1 Department of Health and Environmental Sciences, Xi’an Jiaotong-Liverpool University, Suzhou 215123, China; Yifan.jin14@alumni.xjtlu.edu.cn (Y.J.); Zihan.dong15@alumni.xjtlu.edu.cn (Z.D.); Richard.Carciofo@xjtlu.edu.cn (R.C.); 2 Department of Nutrition and Food Safety, School of Public Health, Xi’an Jiaotong University, Xi’an 710061, China; luoxiaoqin2012@mail.xjtu.edu.cn; 3 Department of Nutrition and Food Hygiene, School of Public Health, Medical College of Soochow University, Suzhou 215123, China; lixinli@suda.edu.cn; 4 Department of Human Nutrition, University of Otago, 9016 Dunedin, New Zealand; Sheila.Skeaff@otago.ac.nz

**Keywords:** iodine, iodine intake, iodine knowledge, young adults, China

## Abstract

Lack of iodine knowledge might be a risk factor for inadequate iodine intake in populations. Therefore, we aimed to determine the relationship between iodine knowledge and intake in young Chinese adults. A cross-sectional study was conducted in Suzhou, China. Iodine intake was assessed using a validated 33-item iodine-specific Chinese food frequency questionnaire (FFQ) and iodine knowledge was determined using a Chinese iodine knowledge questionnaire. A total of 150 participants (mean age 20.3 years) completed the study. The median iodine intake plus iodized salt was 260 μg/d, indicating iodine sufficiency (>150 µg/d). The median iodine knowledge score was 16/24, suggesting a medium level of knowledge. The majority of participants correctly recognized fish and seafood (95%) and iodized salt (83%) as the most important dietary iodine sources. After adjusting for age and sex, studying in the science cluster and having received iodine education were the predictors of having a higher iodine knowledge score, with adjusted odd ratios (OR) of 4.33 (1.49, 12.61) and 2.73 (1.21, 6.14), respectively. In conclusion, young Chinese adults had an adequate iodine intake despite a medium iodine knowledge score. This study provides support that iodine fortification in China has been successful, but further research is required to more fully substantiate this finding.

## 1. Introduction

Iodine is required by the thyroid for the production of thyroid hormones [1]. Therefore, an adequate iodine intake is important to ensure normal thyroid function in the body. According to the WHO/UNICEF/IGN (Iodine Global Network), the recommended iodine intake for adults is 150 µg/d [2]. When iodine intake is <150 µg/d, the risk of iodine deficiency increases [2]. Insufficient iodine intake has been associated with several adverse effects including goiter [3] and a reduced IQ in children [4]. However, excessive iodine intake might also cause thyroid dysfunction in individuals [5].

Dietary iodine intake is regarded as the most direct biomarker of iodine status in populations and individuals [6]. There are several dietary assessment methods that can be used to assess iodine intake in populations and individuals [7]. For example, a 24-h diet recall, weighed food record, and food frequency questionnaire (FFQ). Although both the 24-h diet recall and weighed food record can provide detailed dietary intake information, they are associated with a large respondent burden and are time-consuming [8]. On the other hand, FFQ is relatively easy to administer, allowing the collection of usual iodine intake over a specific period of time [8], which minimizes within-person variability [9].

In response to widespread iodine deficiency across China, in 1994 the Chinese government implemented a mandatory salt iodization program and strengthened a health education strategy, which together aimed to eliminate iodine deficiency disorders [10,11,12]. For example, during the 1980s, the prevalence of goiter in Jiangsu was 26% with a mean urinary iodine concentration (UIC) of 76 μg/L [10]. In addition, over the last two decades, the awareness of iodine deficiency disorders has increased steadily in the Chinese population [11]. To our knowledge, only one study has investigated the association between the level of iodine knowledge and iodine intake, conducted in a group of pregnant Chinese women [13]. There is a lack of data regarding young adults’ knowledge of iodine, particularly those living in the coastal provinces (including Jiangsu Province). Knowledge of iodine is important to ensure that adults have adequate iodine intake, especially young women of childbearing age, because a poor iodine status in pregnancy can adversely affect the child [14]. In addition, chronic iodine deficiency is also associated with a higher risk of thyroid dysfunction in both males and females [2].

Therefore, the aim of this study was to determine the relationship between iodine knowledge and iodine intake as well as to examine predictors of iodine knowledge among young adults in China.

## 2. Materials and Methods

A cross-sectional study was conducted from November 2017 to March 2018 around the campus of Xi’an Jiaotong-Liverpool University (XJTLU) in Suzhou, Jiangsu Province, China. Only adult students of Chinese nationality aged ≥18 years who were able to read, speak, and write Chinese characters were recruited in the study. Convenience sampling was used in the recruitment of participants. The study protocol was approved by the faculty supervisor in accordance with Xi’an Jiaotong-Liverpool University’s Policy for the Procedures for the Ethical Assessment of Undergraduate Final Year Projects Involving Human Research and in compliance with the University’s Research Ethics Sub-Committee Guidelines.

### 2.1. Assessment of Iodine Knowledge Level

Participants were asked to complete a sociodemographic questionnaire that included questions about age, sex, and the presence of thyroid disease. Questions regarding iodine knowledge and attitudes, such as “What is iodine?”, “I think I get enough iodine through diet”, “Which of the following foods are the most important dietary iodine sources?”, and “What is the iodine status in China?”, were also included (Supplementary 1). These validated questions were adapted from previous studies [13,15,16]. In addition, these questions were further validated in our setting and pilot tested in order to check for validity, comprehension, and clarity. An expert opinion (Z.F.M.) was also obtained during the validation process. Participants were asked to recall if they had (a) enough, (b) some, or (c) never received iodine education. The former two options and the last option were classified as “yes” and “no” to iodine education, respectively. The question on iodine education referred to a broad, general question(s) about any kind of iodine education. Participants were not asked to recall a specific iodine education campaign. Iodine knowledge questions were used to calculate the knowledge score of participants with a Cronbach’s alpha of 0.60, which was considered to be acceptable [15]. These scores ranged from 0 to 24, with four different categories (i.e., poor knowledge (0–6 points), low knowledge (7–12 points), medium knowledge (13–18 points), and high knowledge (19–24 points)). As there were only 19 participants with low knowledge scores, participants were placed into two groups based on their knowledge scores as follows: low to medium knowledge (0–18 points) and high knowledge (19–24 points) [15].

### 2.2. Assessment of Dietary Iodine Intake

In addition, participants were also required to complete a 33-item iodine specific FFQ about items that are the main dietary sources of iodine in the Chinese population, such as porridge, buns, noodles, soymilk products, meat, eggs, yoghurt, seafoods, vegetables, fruit, and snacks [17]. The use of iodized salt was also included. Participants were asked to select the appropriate options based on the amount and frequency of consumption of the food over the past month. For each food item, the frequency options included “2 or more times a day”, “once per day”, “5–6 times per week”, “once a week”, “1–3 times per month”, “less than once a month”, or “never”. The iodine intake from each food was determined using the Chinese Food Composition Table [17]. The FFQ had previously been validated in Chinese adults [17] with iodine intake estimated from the FFQ associated with 24-h urinary iodine excretion (UIE) in a group of Chinese adults (r = 0.47, P = 0.009) [17]. Although the correlation was moderate, it was within the acceptable range (0.20–0.49) to indicate agreement between FFQ and UIC [17].

### 2.3. Statistical Analysis

SPSS, Version 23 (IBM Corp., Armonk, NY, USA) was used for the statistical analysis. Descriptive statistics were used to determine the means, standard deviations, and medians for the sociodemographic variables. An unpaired t-test was used to explore whether there was a difference in iodine intake between participants with low to medium and high knowledge levels. Logistic regression was then used to assess the association between iodine knowledge 0 = low and medium scores (knowledge scores ranging from 0–18) and 1 = high scores (knowledge scores ranging from 19–24)) and other variables, after adjusting for age and sex.

## 3. Results

A total of 150 participants (i.e., 57 males and 93 females) were recruited into the study (Table 1). The gender split of those eligible to participate was similar to the ~40:60 male:female ratio study sample and more than 80% of eligible students at this university study in the non-science cluster as in the group sampled. Males had a significantly higher mean age than females (mean ages of 21.1 years and 19.8 years, respectively) (P < 0.001). Most of the participants were undergraduate students (97%) and were from the non-science cluster (such as humanities and social science undergraduate degrees) (83%). Almost one quarter (24%) took dietary supplements but only 11% reported the use of iodine supplements. Only 6% of participants were smokers while 2% had experienced a thyroid condition such as goiter before. More than half of the participants (68%) did not use iodized salt or were unsure whether the type of salt they consumed was iodized when at home.

Nearly half (49%) of the participants had received iodine education. Both males (98%) and females (97%) were confident that they knew what iodine was. However, more males (49%) were more confident that they consumed enough iodine through their diet when compared to females (32%). Overall, around 60% of the participants reported that they were not sure about whether they had consumed enough iodine through their diet. There were 25 students from the science cluster; of these, 52% (n = 13) received iodine education.

Most of the participants correctly recognized fish and seafood (95%) and iodized salt (83%) as the most important dietary sources for iodine (Table 2). However, only 7% of the participants identified milk as an important food source for iodine. In addition, 11%, 10%, and 7% of participants incorrectly identified nuts, meat, and fruits as important food sources for iodine.

In terms of iodine function, more than half of the participants (63%) correctly identified that iodine is important for normal child growth and development. About half of the participants (43%) correctly recognized that iodine is important for maintaining normal metabolism, and 38% of the participants correctly recognized that iodine is important for normal fetal development. Also, more than half of the participants (61%) correctly identified that a very low iodine intake was previously a problem in China but is not a problem now.

The median iodine intake plus iodized salt of the participants was 260 μg/d. In addition, aquatic products, eggs, staple food, meat, and milk, and dairy products were identified as the five main food sources of iodine intake among young Chinese adults (Table 3). There was no association between the use of iodized salt and iodine intake (P = 0.284).

Iodine knowledge scores were calculated and are presented in Table 4. Scores ranged from 8–24 with a median (p25–p75) of 16 (14–19). Most of the participants (69%) had low and medium knowledge levels, and about 31% of the participants had a high iodine knowledge level.

There was no significant association between iodine knowledge levels and iodine intake after adjusting for age (r = 0.06, P = 0.50) (Table 5). Using logistic regression, studying in the science cluster significantly increased the probability of having a higher iodine knowledge level with an adjusted odds ratio (OR) (95% CI) of 4.33 (1.49, 12.61). In addition, having received iodine education also significantly increased the probability of having a higher iodine knowledge level with an adjusted OR (95% CI) of 2.73 (1.21, 6.14).

## 4. Discussion

Iodine deficiency affects about 30% of the total world populations, particularly children and pregnant women [2]. Iodine deficiency has been prevalent in China since the 1930s and about 15% of children had mild mental retardation in many endemic areas [10,18]. As a result, in the 1960s, interventions of salt iodization were implemented to improve the iodine status in these areas [10]. At that time, awareness of the consequences of iodine deficiency was poor in many high-risk regions of China [10]. Therefore, improving health education about the importance of obtaining enough iodine in the diet was an important strategy to eliminate iodine deficiency disorders [19].

We found a relatively high daily iodine intake in these young Chinese adults living in Suzhou, Jiangsu Province, China. Our findings were consistent with the iodine intake reported in other studies conducted in China [17]. Tian et al., using an iodine-specific FFQ, found a mean iodine intake of 261 μg/d in adults (mean age of 22 years) from Tianjin, China [17]. A study by Ding et al. reported that in Zhejiang province, adults living in inland areas had a significantly higher mean iodine intake than those near coastal areas (351 vs. 257 μg/d) [20]. The overall median iodine intake for adults in Zhejiang province was 272 μg/d, and a 24-h diet recall was used to assess the iodine intake [20]. Another study by Zou et al. reported that the iodine intake of adults in Shanghai was 226 μg/d [21]. The relatively high iodine intake among the young Chinese adult population is likely a result of the universal iodized salt (USI) program implemented to ensure that all population groups have sufficient iodine intake [22]. Given the medium level of iodine knowledge of this sample, it is important to know how important iodine education is compared to population prophylaxis by (USI) alone. However, this is probably beyond the scope of this project and it may be impossible to separate the effects of USI and iodine education when introduced simultaneously.

The iodine intake reported by our study was also relatively higher than other studies conducted outside of China [23,24]. New Zealand adults had a median iodine intake of 132 μg/d, which included iodine from iodized salt [23]. A study by O’Kane et al. reported that women of childbearing age living in the UK and Ireland had a median iodine intake of 152 μg/d [24]. Inadequate iodine intake, particularly in women of childbearing age, is of particular concern because iodine deficiency is likely to continue into pregnancy. In addition, given that most pregnancies are unplanned [25], it is important for women of childbearing age to have adequate iodine status. Iodine deficiency during early pregnancy has been associated with several adverse effects in neurocognitive development in children, including verbal IQ and reading scores [4].

To our knowledge, our study was the first study to investigate iodine knowledge and its relationship with iodine intake in a group of young adults in China. Most studies that explored iodine knowledge level only included pregnant and lactating women, who are most vulnerable to iodine deficiency [13,15,24,26]. Very few studies have chosen adults as a target population [16,24,27]. In particular, studies investigating the relationship between iodine knowledge and iodine status in the young adult population are still sparse. In addition, there is no knowledge data prior to 1994 that could be used to see if knowledge has improved since the introduction of iodine education programs. Therefore, we are unable to examine if knowledge has improved since the introduction of iodine education programs.

Our study found a relatively higher iodine knowledge level among young Chinese adults than the findings reported in a group of young Norwegian women [16] (iodine knowledge scores of 16 vs. 14, respectively). The authors reported no difference in iodine knowledge scores between students who were studying health science and other sciences [16]. A study of women of childbearing age conducted in the UK and Ireland by O’Kane et al. reported a positive association between younger age and the iodine knowledge level (P < 0.05) [24]. In addition, a higher education level was associated with a lower risk of inadequate iodine intake (<150 µg/d) [28], because educated adults were more likely to eat foods rich in iodine and were aware of the consequences of iodine deficiency. Our study recruited university students, who had a higher education level. Therefore, a relatively high iodine knowledge level among our participants could be possibly due to a high education level. As we did not determine if a higher iodine knowledge level was due to either the participants’ university education or public health education about iodine, we were unable to confirm if the association between iodine knowledge and education has more to do with the participants’ university education than public health education about iodine. As our lowest score was eight and only 19 participants had low knowledge score (scores ranging from 7–12), we did not have any participants with poor knowledge (i.e., scores <6) (very low iodine intake) to clarify the influence of a very-low iodine knowledge level on iodine intake. Although iodine education may well be a predictor of a higher iodine knowledge level, our study did not further examine if it leads to a behavioral change that is reflected in increased iodine intake. One of the possible reasons why fewer females were confident that they were consuming enough iodine in their diet compared to males maybe that females were under-reporting their food intake.

Our study also found that iodine education was one of the predictors for the iodine knowledge score, which is consistent with the findings from the UK and Ireland [24]. In China, iodine education activities such as promotional events and displays are carried out continuously by the relevant local and regional government authorities in order to increase the awareness and understanding of how to prevent Iodine Deficiency Disorders (IDD) in populations [29,30]. In addition, China has a National IDD Prevention Day on 15 May each year [30]. Social media platforms such as WeiBo and WeChat are also used by the local and regional government authorities to address some misconceptions about the safety of iodized salt [30].

Our study had several strengths, including the use of a validated Chinese FFQ in adults to assess iodine intake [17]. In addition, the collection of habitual iodine intake levels from food sources might serve as a better biomarker of iodine status on an individual level. Although the FFQ is a relatively crude dietary assessment method, it is better suited for categorizing individuals according to different iodine intake levels. In addition, we believed that participants who said no to iodine education might well have had it and just not remembered. Therefore, a low knowledge level might not be due to a lack of education. Future studies should include a more comprehensive questionnaire to investigate the impact of education on iodine intake. We also categorized participants who were unsure whether the type of salt they consumed was iodized as non-iodized salt users, which might underestimate the actual number of participants who were iodized salt users. However, it is possible that some of the participants who were unsure whether the type of the salt they consumed was iodized were using non-iodized salt, because presently, in China, the policy on the availability of non-iodized salt has been relaxed. Consumers can now purchase non-iodized salt from shops or on online e-commerce platforms such as Taobao [29,30].

As iodized salt is usually added to food products during cooking and at the table, an accurate estimation of iodized salt intake is very difficult to assess Moreover, iodine intake is not well measured from dietary assessments due to the difficulties in determining salt intake, and iodized salt can be a major contributor to iodine intake. Therefore, it is very challenging to accurately assess the contribution of iodized salt to the iodine intake. The iodine content in iodized salt in China is set at 25 mg iodine/kg salt (range: 18–33 mg iodine/kg salt).In conducting this study we could not find no knowledge data prior to 1994 that could be used to see if knowledge has improved since the introduction of iodine education programs. Future studies should consider asking participants to recall specific iodine education campaigns because such understanding may then assist in the development of successful educational interventions for at-risk groups, including women of childbearing age. Convenience sampling was used in our study because it is easy and affordable. However, this sampling method is likely to introduce potential bias and our findings were not likely to be representative of all young adults in the region. It is possible that less-educated young adults may have less iodine knowledge, and this may impact on their iodine intake and resultant iodine status. Care needs to be taken to not exaggerate the findings beyond the sample examined if they are not similar to all young adults in the region or beyond. Another limitation of our study was that UIC was not assessed in participants. Measuring UIC is the recommended method of iodine assessment [2]. This is because iodine intake is not well measured from dietary assessment due to difficulties in determining salt intake, and iodized salt is a major contributor to iodine intake [31]. Therefore, it is suggested that future studies should include the collection of blood and urine samples in order to allow for a more comprehensive determination of the iodine status [32].

## 5. Conclusions

In our sample, young Chinese adults had adequate iodine intake and a medium iodine knowledge level. Although our study adds to growing evidence that iodine education and mandatory iodine fortification in China are successful components in the elimination of IDD in populations, our results are not representative of all young adults in China. In addition, it might be the fortification alone which is the important driver for adequate intake. Therefore, future studies should be designed to determine the relationship between the level of iodine knowledge and iodine intake in population sub-groups that are more vulnerable to iodine deficiency.

## Figures and Tables

**Table 1:** Participants’ characteristics.

Characteristics	Total (n = 150)	Males (n = 57)	Females (n = 93)	P-Value
Age (years)	20.3 ± 2.0	21.1 ± 1.9	19.8 ± 1.9	<0.001
Levels of study, n (%)				
Undergraduate	146 (97.3)	55 (96.5)	91 (97.8)	nd ^1^
Postgraduate	4 (2.7)	2 (3.5)	2 (2.2)	
Clusters, n (%)				
Science	25 (16.7)	9 (15.8)	16 (17.2)	<0.001
Non-science	125 (83.3)	48 (84.2)	77 (82.8)	
Supplement use				
Yes	36 (24.0)	18 (31.5)	18 (19.4)	0.132
No	114 (76.0)	39 (68.4)	75 (80.6)	
Smokers	9 (6.0)	5 (8.8)	4 (4.3)	0.271
Have experienced thyroid condition before				
Yes	3 (2.0)	1 (1.8)	2 (2.2)	nd ^1^
No	147 (98.0)	56 (98.2)	91 (97.8)	
Have received iodine education before				
Yes	74 (49.3)	28 (49.1)	46 (49.5)	0.968
No	76 (50.7)	29 (50.9)	47 (50.5)	
Iodized salt users at home				
Yes	48 (32.0)	16 (28.1)	32 (34.4)	0.713
No ^2^	102 (68.0)	41 (71.9)	61 (65.6)	

^1^ nd, not determined due to low cell count. ^2^ Included the participants who were unsure whether the type of the salt they consumed was iodized.

**Table 2:** Young Chinese adults’ knowledge regarding dietary iodine sources, iodine functions, and the national iodine status.

Iodine Knowledge	Total (n = 150)	Males (n = 57)	Females (n = 93)	P-Value
Most important dietary iodine sources, n (%)				
Meat	15 (10)	5 (8.8)	10 (10.8)	0.695
Milk	10 (6.7)	5 (8.8)	5 (5.4)	0.425
Fruits	11 (7.3)	4 (7.0)	7 (7.5)	0.907
Fish and seafood	142 (94.7)	55 (96.5)	87 (93.5)	0.423
Bread	5 (3.3)	1 (1.8)	4 (4.3)	nd ^1^
Vegetable oil	1 (0.7)	1 (1.8)	0 (0.0)	nd ^1^
Nuts	16 (10.7)	9 (15.8)	7 (7.5)	0.112
Iodized salt	125 (83.3)	51 (89.5)	74 (79.6)	0.114
Do not know	1 (0.7)	0 (0.0)	1 (1.1)	nd ^1^
Iodine is important for, n (%)				
Normal child growth and development	95 (63.3)	34 (59.6)	61 (65.6)	0.464
Preventing blindness	21 (14.0)	9 (15.8)	12 (12.9)	0.621
Normal fetal development	57 (38.0)	20 (35.1)	37 (39.8)	0.565
Strength in teeth and skeleton	31 (20.7)	13 (22.8)	18 (19.4)	0.612
Maintaining normal metabolism	65 (43.3)	29 (50.9)	36 (38.7)	0.144
Preventing spina bifida	23 (15.3)	8 (14)	15 (16.1)	0.730
Do not know	10 (6.7)	3 (5.3)	7 (7.5)	0.584
Iodine status in China, n (%)				
Too low intake is a current problem	20 (13.3)	9 (15.8)	11 (11.8)	0.488
Too high intake is a current problem	20 (13.3)	7 (12.3)	13 (14)	0.767
Too low intake was a problem earlier, not now	91 (60.7)	39 (68.4)	52 (55.9)	0.148
Do not know	20 (13.3)	2 (3.5)	18 (19.4)	0.006

^1^ nd, not determined due to low cell count.

**Table 3:** Mean daily iodine intake from foods only in young Chinese adults.

Food Categories	Examples of Foods	Contribution to Iodine Intake (%)
Staple food	Steamed bread, rolls, and cakes	1.7
	Rice and rice products	5.9
	Buns, dumplings, and wonton	0.7
	Noodles, vermicelli, and ramen	2.8
	Porridge	1.0
Total		12.2
Beans and bean products	Beans	0.2
	Soybean milk	3.0
	Bean product	0.8
Total		4.0
Meat	Pork	0.1
	Beef and mutton	0.3
	Chicken	0.3
	Meat product	8.6
Total		9.3
Eggs	Egg	7.3
	Duck egg	4.0
	Century egg	1.7
	Salted duck egg	3.6
Total		16.6
Milk and dairy products	Milk	14.0
	Yogurt	3.6
Total		17.6
Aquatic products	Freshwater fish	1.5
	Sea fish	3.9
	Shrimps	3.7
	Kelp	2.7
	Seaweed	17.7
Total		29.5
Vegetables	Fresh vegetables	3.3
	Lotus root	0.9
	Agarics and snow fungus	0.3
	Small pickles	0.2
Total		4.7
Fruits and nuts	Fruits	5.0
	Peanut	0.1
	Red dates	0.0
	Other nuts	0.1
Total		0.2
Snacks	Cookies	0.4
	Cake	0.6
Total		1.0

**Table 4:** Iodine knowledge levels in young Chinese adults.

Iodine Knowledge Score	Males (n = 57)	Females (n = 93)	P-Value	Total (n = 150)
Total score	17 (14–19) ^1^	16 (13–19)	0.194	16 (14–19) ^1^
Score categories, n (%)				
*Low to medium*	39 (68.4%)	65 (69.9%)	0.096	104 (69.4)
*High*	18 (31.6%)	28 (30.1%)		46 (30.7)

^1^ Median (p25–p75) (all such values).

**Table 5:** Iodine intake of young Chinese adults with different iodine knowledge levels.

	Iodine Intake (μg/d)		P-Value
	Low and Medium Iodine Knowledge Level (n = 104)	High Iodine Knowledge Level (n = 46)	
**Total (n = 150)**	267 (237, 298) ^1^	255 (235, 281)	0.713
Males (n = 57)	265 (236, 318)	255 (246, 298)	0.427
Females (n = 93)	267 (235, 287)	256 (233, 279)	0.738

^1^ Median (p25–p75) (all such values).

## Supplementary Materials

The following are available online at https://www.mdpi.com/2254-9625/10/1/40/s1, Questionnaire S1 sociodemographic questionnaire, iodine knowledge and attitudes.
