# Supplementary material for: Adequate Iodine Intake among Young Adults in Jiangsu Province, China Despite a Medium Iodine Knowledge Score
Source: Eur J Investig Health Psychol Educ. 2020 Mar 9;10(1):554–63. doi: 10.3390/ejihpe10010040 (PMC8312987; doi:10.3390/ejihpe10010040)
Supplement: Supplementary file 1 [file ejihpe-10-00040-s001.pdf]

Questionnaires

A. Socio-demographic information

1. Gender
   A. Male   B. Female

2. Age: ____

3. Which year do you belong to in XJTLU?
   A. Year 1   B. Year 2   C. Year 3   D. Year 4   E. Undergraduate

4. Which cluster do you belong to in XJTLU?
   A. Science   B. Industrial Technology   C. Business   D. Build Environment
   E. Mathematical Sciences   F. Humanities and Social Sciences

5. Which province are you from？ __________

6. Do you take any supplements?
   A. Yes   B. No

   If you take supplements, then do you take iodine supplements?
   A. Yes   B. No, I take ________________

7. Are you a smoker?
   A. Yes   B. No

8. Have you ever been diagnosed with a thyroid condition?
   A. Yes
   B. No

9. Have you ever received any iodine education?
   A. Received enough iodine education
   B. Received some iodine education
   C. Never received any iodine education

10. Do you think the salt you take is iodized?
    A. Yes
    B. No
    C. Not sure

B. Iodine attitude

1. Do you know what iodine is?

   A. Yes B. No

2. I think I get enough iodine through my diet.

   A. Agree B. Disagree C. Don't know

C. Iodine knowledge

The following three questions are multiple choice questions; you can choose one or more than one answer.

1. Which of the following foods are the most important dietary iodine sources? (Multiple choice)

A. Meat
B. Milk
C. Fruit
D. Fish and seafood
E. Bread
F. Vegetable oil
G. Nuts
H. Iodized salt
I. Don't know

2. Iodine is important for （Multiple choice）

A. Normal child growth and development
B. Preventing blindness
C. Normal fetal development
D. Strength in teeth and skeleton
E. Maintaining normal metabolism
F. Preventing spina bifida
G. Don't know

3. What is the iodine status in China? (Multiple choice)

A. Too low intake is a current problem
B. Too high intake is a current problem
C. Too low intake was a problem earlier, not now
D. Don't know
